# Do Probiotics in Pregnancy Reduce Allergies and Asthma in Infancy and Childhood? A Systematic Review

**DOI:** 10.3390/nu14091852

**Published:** 2022-04-28

**Authors:** Alexander S. Colquitt, Elizabeth A. Miles, Philip C. Calder

**Affiliations:** 1 School of Human Development and Health, Faculty of Medicine, University of Southampton, Southampton SO16 6YD, UK; asc2g19@soton.ac.uk (A.S.C.); e.a.miles@soton.ac.uk (E.A.M.); 2 NIHR Southampton Biomedical Research Centre, University Hospital Southampton NHS Foundation Trust and University of Southampton, Southampton SO16 6YD, UK

**Keywords:** pregnancy, infancy, childhood, immune development, probiotic, microbiota, allergy, asthma, eczema, atopic dermatitis

## Abstract

The maternal immune system is very important in the development of the foetal immune system. Probiotics have been shown to help regulate immune responses. Therefore, it is possible that the administration of probiotics to pregnant women could influence the development of the foetal immune system, reducing the likelihood of infants and children developing an allergic condition. The aim of this research was to conduct a systematic review to determine whether administering probiotics to pregnant women can reduce the incidence of allergic disease in their children. Medline, CINAHL and Embase databases were searched for randomised controlled trials (RCTs) that compared supplementation of probiotics to pregnant women to a placebo control and recorded the presentation of allergic conditions in their children. Data extracted from the study reports included their characteristics and findings. Study quality and risk of bias were assessed. From a total of 850 articles identified in the search, 6 were eligible for inclusion in this review. Two studies found no effect of maternal probiotics on the outcomes measured, two studies found that the incidence of eczema or atopic dermatitis (AD) was reduced by maternal probiotics, one study found no effect on the overall incidence of atopic sensitisation, but a reduction in a subgroup of children at high hereditary risk of allergic disease, and one study found no effect in an intention to treat analysis, but a reduction in AD in complete case analysis. The results of these studies are inconsistent but demonstrate that probiotics may have the potential to reduce infant allergies when administered prenatally, particularly in children at high risk of allergy development. There is a need for further larger-scale studies to be performed in order to provide a more definitive answer. Such studies should focus on at-risk groups.

## 1. Introduction

The incidence of allergic disorders such as atopic dermatitis (AD, also called atopic eczema), allergic rhinoconjunctivitis (ARC), food allergies and asthma has increased over the last decades in both developed and developing countries [1,2,3,4,5]. Allergy is caused when the immune system actively responds to otherwise harmless antigens [6], and these antigens are referred to as allergens. Allergic reactions can be categorised into two types: immunoglobulin E (IgE)-mediated and non-IgE-mediated. IgE-mediated reactions include ARC, food allergies and allergic asthma, and are generally characterised by the T helper 2 (Th2) cell inflammatory pathway [6,7]. The initial setting up of IgE-mediated allergic disease occurs when an infant is first sensitised to an allergen [6]. There is evidence that such predisposition to allergic disease occurs in foetal life, i.e., before birth [8,9]. Eczema (i.e., AD) is the first manifestation of allergic disease in infants, followed by food allergy, asthma and allergic rhinitis, and asthma may not manifest until 5 years of age and allergic rhinitis until 7 years of age. This progressive development of allergic disease is referred to as the “atopic march” [10,11].

The gut microbiota is believed to be important in immune development and determining allergy risk [12]. It is widely considered that the human foetus is sterile when in utero, although recent studies have challenged this [13]. Nevertheless, neonates are exposed to microbes during and after the birthing process from their mother and surroundings [14]. The mode of delivery has a significant effect on the microbes that are able to colonise the gut, and microbes are also transferred from mother to infant via kissing, suckling and hugging directly after birth [14]. Post-partum, infants are exposed to new microbes via food (e.g., in mother’s breastmilk) and by entering new environments. The infant microbiota seems to be essential in the development of a mature immune system, and its manipulation could alter the course of development of allergic disease [15]. Older studies identified that infants raised on farms had a lower risk of allergic disease [16,17], and these observations gave rise to the “hygiene hypothesis” [18] that linked early exposure to microbes to more optimal immune maturation and in turn to reduced allergy risk. Furthermore, infants who were born via a natural birth instead of caesarean section, exclusively breastfed or not exposed to antibiotic treatment had more diverse microbiota, which also correlated with a lower risk of developing allergic disease [19]. Together, these observations suggest that strategies to manipulate the gut microbiota may be a means for lowering the risk of allergic disease. In this context, probiotics are a way of manipulating the infant microbiota to increase its diversity for the purpose of preventing allergies [15,20]. There are many different probiotic species, but lactobacilli and bifidobacteria are amongst the most common [21]. Both have been studied extensively in the context of allergy and have been associated with reductions in rates of AD, food allergy, ARC and asthma [20,22]. These bacteria are shown to modulate the host’s Th1/Th2 balance by producing cytokines that promote the Th1 pathway, therefore suppressing the Th2 pathway that is associated with allergy development [20,22].

Several studies have linked probiotics with the primary prevention of allergies and allergic diseases, especially when taken by the children themselves [23,24,25], but there are fewer studies that look into the effect of manipulation of the pregnant mother’s microbiota and allergy risk of the child. Randomised controlled trials (RCTs) that have been conducted in the field have produced inconsistent results, and therefore systematic reviews are needed to summarise the evidence and try to arrive at a clearer view of the findings and to identify knowledge gaps and research priorities. One such review was recently published on this topic [25]. The authors concluded that some probiotic mixtures do “probably reduce the risk of developing atopic dermatitis compared with placebo” [25]; however, the review included many studies in which the probiotic was administered to both the mother and the infant, as well as focusing on AD as the primary outcome.

The aim of the current review is to assess the impact of prenatal probiotic use (i.e., administering probiotics to the mother only) on the development of a wider range of allergic conditions, including both AD and asthma.

## 2. Materials and Methods

### 2.1. Overview

This systematic review was conducted according to the “Preferred Reporting Items for Systematic review and Meta-Analysis” (PRISMA) guidelines [26] and the reporting herein is consistent with these. The review was not registered as it was performed for educational purposes and a formal protocol was not prepared. The PICO (Patient or population, Intervention, Comparison and Outcome) approach was utilised to identify search terms. The population group was pregnant women and their offspring, the intervention was any probiotic, the comparison was between groups of offspring of mothers who received probiotics or who received a control and the outcome measure was any outcome related to allergy, including asthma.

### 2.2. Literature Search

The following databases were searched for relevant literature: Ovid MEDLINE (1946 to week 2 of September 2021), EMBASE (1974 to 22 September 2021) and CINAHL. Free-text searches, using the terms: ‘pregnan*’, ‘prenatal’, ‘pre-natal’, ‘pregnant women’, ‘mother’, ‘probiotic’, ‘allerg*’, ‘hypersensitiv*’, ‘dermatitis’, ‘asthma*’, ‘raised IgE’, ‘atop*’, ‘eczema’, ‘skin prick test’, ‘SPT’, ‘child*’, ‘offspring’ and ‘infan*’, were used.

### 2.3. Study Selection

Studies were selected for this systematic review based on the following inclusion criteria: must be an RCT, must have compared a probiotic treatment to a control group, mothers must have received the probiotic during pregnancy or pregnancy and lactation, a measure of allergy must have been reported in the children, published as a full research paper and published in the English language. Exclusion criteria included: combined use of probiotics with another intervention, use of synbiotics and probiotics administered to the children.

### 2.4. Data Extraction

Data from the studies that were extracted included sample size, probiotic used, type of control, duration of treatment, outcomes measured, test results for those outcomes and the conclusions drawn from those results.

### 2.5. Quality Assessment

The studies included in this systematic review were assessed for bias using the Cochrane Risk of Bias 2 tool [27] and assessed for quality using the Jadad quality scale [28]. The Cochrane Risk of Bias tool is a system that assesses whether a study holds a high, medium or low risk of bias by asking a series of questions over 5 domains in which bias may arise. The Jadad quality scale is a 0–5-point scale (0 being lowest quality, 5 the highest quality) in which points are awarded or deducted for a study based on a series of 7 questions.

## 3. Results

### 3.1. Search Results

**Figure 1:**
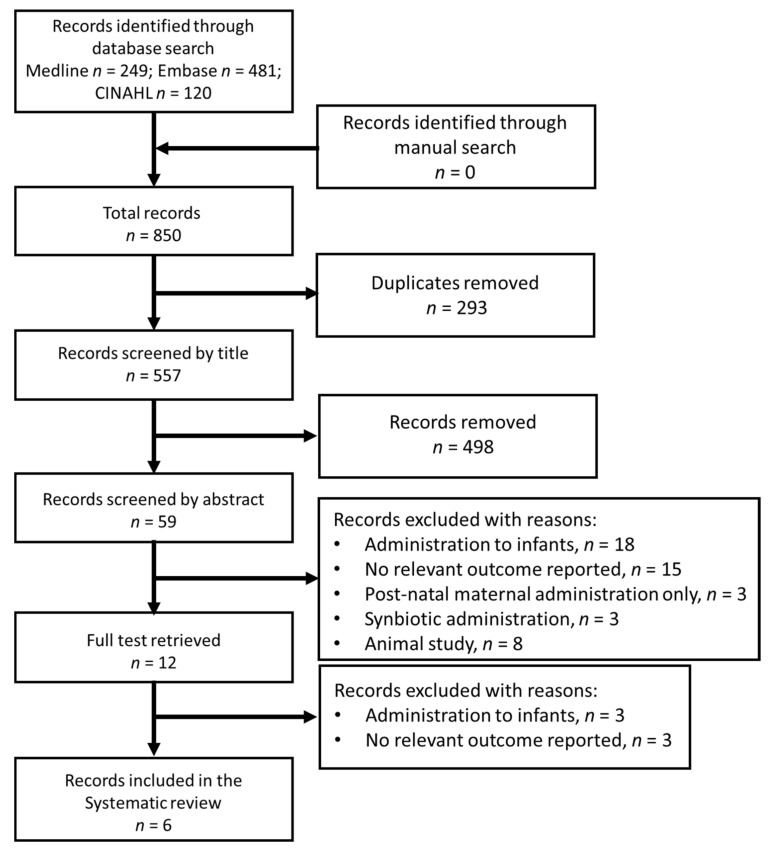
Flow diagram summarizing the identification and selection of articles for inclusion in the review.

The search of 3 databases returned a total of 850 records, with no additional records found with a manual search (Figure 1). Of these records, 293 were duplicates, leaving 557 records. After screening by title, 498 of these records were removed, and a further 47 were removed after screening by abstract, leaving 12 records. The full articles were retrieved for these records and 6 were removed for the following reasons: administration of probiotics to the infants (*n* = 3) and no relevant outcome measured in infants (*n* = 3). 

### 3.2. Characteristics of the Included Studies

Six papers were included [29,30,31,32,33,34]. These represent five separate trials because two papers were published from the same trial but reporting outcomes at two different periods of follow-up [30,33]. Key characteristics of the trials included in this review are presented in Table 1. Initial points to note are that 3 out of the 5 trials (4 out of 6 included articles) involved the administration of a common probiotic species: *Lactobacillus rhamnosus* GG [29,31,33], 4 studies (5 articles) involved post-natal (as well as prenatal) administration of probiotics to the mother [30,31,32,33,34] and 1 study stopped the intervention at the time of delivery [29]. 

Boyle et al. [29] randomised 250 pregnant women (212 of whom completed the trial) to receive either *Lactobacillus rhamnosus* GG intervention or a maltodextrin placebo from 36 weeks of gestation to delivery. The children were assessed for eczema as the trial’s primary outcome, along with eczema severity, whether IgE-associated, and also atopic sensitisation in the form of a skin prick test (SPT) at 3 follow-up sessions at ages 3, 6 and 12 months.

Dotterud et al. [30] randomised 415 women (278 of whom completed) to receive either a probiotic milk containing a mixture of *Lactobacillus rhamnosus* GG, *Bifidobacterium animalis* subsp. *lactis* BB12 and *Lactobacillus acidophilus* La-5, or a heat-treated sterile milk placebo. The treatment lasted from 36 weeks of gestation to 3 months post-natal. The primary outcome was the development of atopic disease within two years, so children were assessed for AD, asthma and ARC at a two-year follow-up session, and the children were also assessed for atopic sensitisation in the form of a SPT.

Huurre et al. [31] randomised 140 pregnant women to receive a probiotic mixture of *Lactobacillus rhamnosus* GG and *Bifidobacterium lactis* BB12, or a microcrystalline cellulose and dextrose anhydrate placebo from the 1st trimester until the end of exclusive breastfeeding. The children were followed up 3 times at ages 1, 6 and 12 months and were assessed for atopic sensitisation by SPT.

Rautava et al. [32] randomised 241 women into 3 groups—2 intervention groups and 1 placebo. The intervention groups received a probiotic mix of either *Lactobacillus rhamnosus* LPR and *Bifidobacterium longum* BL999 or *Lactobacillus paracasei* ST11 and *Bifidobacterium longum* BL999 in the form of a tablet which also contained vitamins and minerals. The placebo group received the same multi-vitamin and mineral tablet but without any probiotic bacteria. The intervention lasted from 2 months prenatal until 2 months post-natal. The children were assessed at 5 follow-up sessions at 1, 3, 6, 12 and 24 months, primarily for eczema, and a SPT was also performed to test for atopic sensitisation.

Simpson et al. [33] performed a 6-year follow-up of the Pro-PACT study completed by Dotterud et al. [26], where 281 participants completed the follow-up. The primary outcome was any atopic disease within 6 years, and the children were assessed for AD, asthma (within the last year), ARC as well as atopic sensitisation by SPT.

Wickens et al. [34] randomised 423 pregnant women to receive either *Lactobacillus rhamnosus* HN001 or a maltodextrin placebo. The intervention was administered from 14 to 16 weeks of gestation up until 6 months post-partum. The children were assessed at 6 and 12 months for eczema, and secondary outcomes were eczema severity on the SCORAD scale, wheeze and atopic sensitisation by SPT.

### 3.3. Effects of Probiotics on Infant Eczema and AD

A full description of the findings reported in the six included papers can be found in Table 2. A diagnosis of eczema or AD was reported in five of the six papers included [29,30,32,33,34] and was the primary outcome in three papers [29,32,34]. Boyle et al. [29] found that the probiotic intervention had no significant effect on eczema diagnosis at one year, including IgE-associated eczema, and had no effect on eczema severity. Dotterud et al. [30] reported a reduction in the overall incidence of AD at two years, and this effect was greatest in the non-IgE-associated AD subgroup, as there was no effect in the IgE-associated subgroup. Rautava et al. [32] found that both probiotic interventions had a very similar effect, and both significantly reduced the rate of eczema at two years in the children compared to the placebo. In their follow-up of the Pro-PACT study [30], Simpson et al. [33] found that there was no effect on AD at six years in the intention to treat analysis; however, there was a significant reduction in AD in the complete case analysis. Adjustment of the results for family history, child sex and siblings did not alter these findings [33]. Wickens et al. [34] found no effect on the incidence of eczema or its severity at one year.

### 3.4. Effects of Probiotics on Infant Asthma

Asthma was reported in two papers [30,33], both from the same study, and wheeze was reported in one paper [34]. Dotterud et al. [30] reported that there was no effect on asthma in either intention to treat or complete case analysis. Simpson et al. [33] reported in their follow-up to Dotterud et al. [30] that the intervention had no effect on incidence of asthma. Wickens et al. [34] also reported that probiotic intervention had no effect on wheeze.

### 3.5. Effects of Probiotics on Infant Atopic Sensitisation

Atopic sensitisation determined by a positive SPT was assessed in all papers included and was the primary outcome of Huurre et al. [31]. In that study, there was no reduction in the overall incidence of atopic sensitisation; however, there was a reduction in a subgroup of children who were at a high hereditary risk of allergic disease (due to maternal sensitisation). There was no effect of probiotics in pregnancy on atopic sensitisation in any other paper [29,30,32,33,34].

### 3.6. Effects of Probiotics on Infant ARC

Incidence of ARC was measured in the Dotterud et al. Pro-PACT study [30] and the Simpson et al. six-year follow-up of this study [33]. Dotterud et al. [30] reported only one case in each group. Simpson et al. [33] reported no effect of maternal probiotics on ARC.

### 3.7. Quality and Risk of Bias Assessment

Five of the six included papers [29,30,32,33,34] were assessed as having a low risk of bias using the Cochrane risk of bias assessment (Table 3) and were awarded 5/5 on the Jadad quality scale (Table 4). The study by Huurre et al. [31] was given a high risk of bias due to the fact that the methods of randomisation and of group assignment concealment were not disclosed, and also because there was no explanation for the participants who dropped out of the trial or for variable sample sizes in the results. The same paper [31] was also given a score of 3 on the Jadad scale due to there being no description of the method of randomisation of dropouts/withdrawals.

## 4. Discussion

This systematic review found limited evidence in support of the hypothesis that probiotics in pregnancy will reduce risk of allergic disease in the children; however, some studies produced findings in support of this hypothesis. Therefore, overall, the findings are inconsistent. This may be due to differences among the studies, such as the probiotic used, when the intervention was started, the duration of the intervention and the risk of the child. For example, Huurre et al. [31] found that maternal probiotics caused a significant reduction in risk of AD at 12 months in infants at high hereditary risk due to maternal allergic sensitisation, but no effect of maternal probiotics in the cohort of infants as a whole. This systematic review included six papers from five randomised, double-blind, placebo-controlled trials of probiotic supplements administered to pregnant and nursing mothers for the prevention of atopic disease and allergy [29,30,31,32,33,34]. The studies assessed the use of seven different strains of probiotics (*Lactobacillus rhamnosus* GG, *Bifidobacterium animalis* subsp. lactis BB12, *Lactobacillus acidophilus* La-5, *Lactobacillus rhamnosus* LPR, *Bifidobacterium longum* BL999, *Lactobacillus paracasei* ST11, *Lactobacillus rhamnosus* HN001), which were used alone in two studies [29,34] and in combination in three [30,31,32,33]. Probiotics were administered from 36 weeks of gestation to delivery (approximately 4 weeks) [29], 36 weeks of gestation to 3 months post-natal (approximately 16 weeks) [30,33], 1st trimester to end of exclusive breastfeeding (approximately 50 weeks) [31], 2 months prenatal to 2 months post-natal (approximately 16 weeks) [32], or 14–16 weeks of gestation to 6 months post-partum (approximately 50 weeks) [34]. Studies reported on the presentation of AD/eczema [29,30,32,33,34], atopic sensitisation [29,30,31,32,33,34], asthma [30,33], wheeze [34] and ARC [30,33], and three studies followed up infants within one year [29,31,34], and two within two years [30,32]. Simpson et al.’s study [33] was a six-year follow-up of Dotterud et al.’s work [30]. None of the included studies reported any adverse effects experienced by mothers or infants during probiotic supplementation, which supports previous research into the safety of probiotic use during pregnancy and lactation [35].

Five of these papers [29,30,31,33,34] were included in a recently published systematic review and meta-analysis that included twenty-one studies of probiotics administered pre- or postnatally, including in infants [25], which concluded that “[certain probiotic preparations] probably reduce the risk of atopic dermatitis based on low-quality evidence compared with placebo when given to infants” [25]. Four of the papers [29,31,32,33] were also included in an earlier review which assessed a total of seventeen studies [23], concluding that “strain-specific sub-meta-analyses showed that probiotic mixtures were effective in reducing the incidence of eczema, while no effect was documented for products containing lactobacilli or bifidobacteria alone” [23]. The reason for the smaller number of included papers in the present review is that the focus was on the administration of probiotics to the mother only, whereas a large proportion of the current literature includes administration to infants, too. Despite the differences in these two previous reviews, the conclusion of this review remains qualitatively similar: that although the results of RCTs are inconsistent, some show that maternal probiotic supplementation can reduce the prevalence of infant AD and eczema, but that there is no effect on other atopic conditions such as asthma and ARC.

Three out of six studies concluded that the incidence of AD or eczema was reduced by maternal probiotics [30,32,33], but there were no effects reported on ARC or asthma. One study [31] described no overall effect on atopic sensitisation, but a reduction in risk among a subgroup of children at high hereditary risk of atopic disease. When analysing the results with regard to the species of probiotic used, in the two studies where lactobacilli were assessed alone [29,34], there were no effects found on infant atopic disease. There were no studies that administered bifidobacteria alone. Three studies (four papers) assessed the administration of different combinations of probiotics [30,31,32,33], all of which included both a lactobacillus and a bifidobacterium strain. All of these combinations showed some form of reduction in the rate of infant atopy—either in AD or atopic sensitisation—showing that perhaps bifidobacteria or a combination of probiotic species are more effective at preventing atopic disease than lactobacilli alone. 

The studies included in this review were of varying duration: one of the trials administered probiotics from 36 weeks of gestation up until delivery [29]. This was the only study not to continue probiotic use after delivery and yielded no significant difference in the rates of eczema in infants between the probiotic and placebo groups. The study by Dotterud et al. [30] and it’s follow-up by Simpson et al. [33] also administered a probiotic from 36 weeks of gestation, but continued administration until 3 months post-natal and found a reduction in AD. Two studies [31,34] started probiotic administration at the end of the first trimester: Huurre et al. [31] stopped administration at the end of exclusive breastfeeding (approximately six months) and Wickens et al. [34] stopped at six months post-natal. Rautava et al. [32] administered the probiotic formulas from two months prenatal to two months post-natal and reported that infants in both probiotic groups had significantly lower rates of atopic disease than the placebo groups. These results suggest that a supplementation period of approximately 16 weeks can be effective [30,32,33] and that the post-natal period, including the period of breastfeeding, may be essential to the effects of probiotics on allergy risk. When comparing the studies by duration of follow-up, the studies that reported results after two years [30,32] seemed to show a greater reduction in rates of atopic disease than those that reported after one year [29,31,34], although this may be due to differences in the probiotic species used and the duration of administration. Interestingly, the six-year follow-up [33] to Dotterud et al. [30] reported a less significant reduction in atopic disease between the treatment groups than the original two-year follow-up. This may be because other factors such as lifestyle begin to have a greater effect as the child gets older; in other words, the effect of the probiotic may wear-off over time.

The results of risk of bias and quality assessments showed that all but one of the included studies carried a low risk of bias. The study by Huurre et al. [31] was deemed to carry a high risk of bias due to the fact that the method of randomisation of mother/infant pairs to probiotic and placebo groups and the method of blinding were not disclosed and the number of participants who dropped out of the study was not clearly reported. The report by Huurre et al., was the smallest of the 6 included studies, with 140 mother/infant pairs being randomised, and it concluded that there was no overall effect of maternal probiotic supplementation on atopic sensitisation in their children; however, there was a reduced risk among a subgroup of children at high hereditary risk of atopic disease. This suggests that probiotics may have a greater effect in higher-risk than lower-risk infants/children.

The strength of this review is the inclusion of trials in which probiotics were administered only to pregnant mothers and not infants. This allowed the analysis of more homogeneous studies when compared to other reviews which included trials that administered probiotics, prebiotics and synbiotics to both mothers and their infants. Another strength of this review is the assessment of only double-blind RCTs, which minimised any bias that could arise during a study. This risk of bias was further minimised by the use of the Cochrane risk of bias tool and Jadad quality assessment to identify any errors in conducting or reporting the trials. One limitation of the review, however, comes from a potential for publication bias due to the fact that RCTs that were not published in the English language were excluded and so some relevant studies may have been missed. Inclusion of other studies was maximised by conducting a manual literature search of reference lists of included papers and other systematic reviews, which found no additional studies. There is potential that studies may have been completed within the field but not published, and so the data were not available. Other important limitations of this review are the relatively small number of studies available for analysis and that there were not any two studies that reported on the administration of the same probiotic. Finally, it is important to note that the studies included in this review were of modest sample size for reporting on clinical outcomes, although four out of the five studies (five out of the six papers) [29,30,32,33,34] performed sample size estimates and recruited women according to those. 

The outcomes of this review show that there is some potential for probiotics to be used by mothers during pregnancy and after delivery as a preventative measure for AD in infants. The evidence suggests that the most effective method is to administer lactobacilli and bifidobacteria strains in combination for the months leading up to delivery and for 3–6 months post-partum. Future research should aim to compare the efficacies of different probiotic strains, as well as determine the optimal time to start the use of probiotic supplements and the duration of their use to achieve consistent long-term benefits. Finally, a comparison of effects of maternal probiotics between high- and low-risk groups should be performed.

## 5. Conclusions

The results from the five studies reported in the six papers were inconsistent but demonstrated that probiotics may have the potential to reduce the risk of infant AD or eczema when administered to mothers both during pregnancy and for a period of 3–6 months post-partum. In particular, treatment containing a combination of lactobacilli and bifidobacteria probiotic strains may be effective, especially if the child is at a high hereditary risk of developing an allergic condition. There is a need for further larger-scale studies to be performed in order to provide a more definitive answer. Such studies should focus on at-risk groups.

## Figures and Tables

**Table 1:** Characteristics of the studies included in the review.

Reference	Country Where Trial Conducted	Number of Mothers Randomised/ Completed	Probiotic Used	Control	Time of Treatment (Start, End)	Outcomes Assessed	Age at Follow-Up
Boyle et al. [29]	Australia	250/212	*L. rhamnosus* GG	Maltodextrin	36 weeks of gestation, delivery	Primary outcome: Eczema during 1st year Secondary outcomes: Allergic sensitisation, IgE-associated eczema, Eczema severity	3, 6, 12 months
Dotterud et al. [30]	Norway	415/278	Milk with: *L. rhamnosus* GG, *B. animalis* BB12 and *L. acidophilus* La-5	Heat-treated sterile milk	36 weeks of gestation, 3 months post-natal	Primary outcome: Atopic disease in first 2 years AD, Asthma, ARC Secondary outcome: Atopic sensitisation, IgE-associated AD, Non-IgE-associated AD	2 years
Huurre et al. [31]	Finland	140/NA	*L. rhamnosus* GG and *B. animalis* BB12	Microcrystalline cellulose and dextrose anhydrate	1st trimester, end of exclusive breastfeeding	Atopic sensitisation (skin prick test) at 12 months	1, 6, 12 months
Rautava et al. [32]	Finland	241/205	Multivitamin andmineral supplement + EITHER *L. rhamonosus* LPR and *B. longum* BL999 OR *L. paracasei* ST11 and *B. longum* BL999	Multivitamin and mineral supplement	2 months prenatal, 2 months post-natal	Primary outcome: Eczema by age 2 years Secondary outcome: Atopic sensitisation (skin prick test)	1, 3, 6, 12, 24 months
Simpson et al. [33]	Norway	415/281	Milk with: *L. rhamnosus* GG, *B. animalis* BB12 and *L. acidophilus* La-5	Heat-treated sterile milk	36 weeks of gestation, 3 months post-natal	Primary outcome: Atopic disease in first 6 years AD, Asthma, ARC Secondary outcome: Atopic sensitisation	6 years
Wickens et al. [34]	New Zealand	423/403	*L. rhamnosus* HN001	Maltodextrin	14–16 weeks of gestation, 6 months post-natal	Primary outcome: Eczema within 12 months Secondary outcomes: SCORAD > 10, Wheeze, Atopic sensitisation	6, 12 months

NA indicates not available.

**Table 2:** Findings of the studies included in the review.

Reference	Outcomes Assessed	Effect of Probiotic			Conclusion
Boyle et al. [29]	Primary outcome: Eczema during 1st year Secondary outcomes: Allergic sensitisation, IgE-associated eczema, Eczema severity	Risk difference: −4.7% (−16.9, 7.4) 0% (−12.7, 12.8) −1.1% (−11.6, 9.5) N/A			No effect for any outcome
Dotterud et al. [30]	Primary outcome: Atopic disease in first 2 years AD, Asthma, ARC Secondary outcome: Atopic sensitisation IgE-associated AD Non-IgE-associated AD	ITT analysis OR 0.51 (0.30, 0.87) 0.68 (0.26, 1.80) N/A 1.45 (0.46, 4.59) 0.90 (0.37, 2.17) 0.43 (0.23, 0.81)	Complete case series analysis OR 0.51 (0.30, 0.87) 0.66 (0.26, 1.66) N/A 1.19 (0.35, 4.01) 0.91 (0.36, 2.31) 0.43 (0.23, 0.83)	Per protocol analysis OR 0.47 (0.26, 0.85) N/A N/A N/A 0.92 (0.36, 2.36) 0.37 (0.18, 0.77)	Reduced incidence of AD. No effect on asthma or ARC. No effect on atopic sensitisation. Reduced incidence of non-IgE-associated AD
Huurre et al. [31]	Atopic sensitisation (skin prick test) at 12 months	All infants OR 0.92 (0.45, 1.0) Infants at high hereditary risk OR 0.34 (0.13, 0.88)			No effect on overall incidence of atopic sensitisation. Reduced incidence in infants at high hereditary risk
Rautava et al. [32]	Primary outcome: Eczema by age 2 years Secondary outcome: Atopic sensitisation (skin prick test)	OR LPR + BL999 0.17 (0.08, 0.35) ST11 + BL999 0.16 (0.08, 0.35) LPR + BL999 0.81 (0.36, 1.76) ST11 + BL999 0.99 (0.46, 2.13)			Reduce risk of eczema but no effect on sensitisation
Simpson et al. [33]	Primary outcome: Atopic disease in first 6 years AD, Asthma, ARC Secondary outcome: Atopic sensitisation	ITT analysis (OR) 0.64 (0.39, 1.07) 1.68 (0.21, 13.20) 1.19 (0.66, 2.16) 1.11 (0.62, 1.96)		Complete case analysis (OR) 0.48 (0.25, 0.92) 3.25 (0.33, 31.6) 1.22 (0.64, 2.37) 1.25 (0.62, 2.54)	No effect on AD in ITT analysis but less AD in complete case analysis. No effect on asthma, ARC or atopic sensitisation
Wickens et al. [34]	Primary outcome: Eczema within 12 months Secondary outcomes: SCORAD > 10, Wheeze, Atopic sensitisation	Hazard ratio 0.83 (0.53, 1.29) 0.95 (0.69, 1.31) 0.89 (0.66, 1.20) 1.02 (0.63, 1.64)			No effect on any outcome

Figures in parentheses represent the 95% confidence interval. ITT, intention to treat; N/A not available; OR, odds ratio.

**Table 3:** Bias assessment of included studies based upon the Cochrane risk of bias tool. Green indicates low risk, orange indicates moderate risk and red indicates high risk.

Reference	Domain 1: Randomisation Process	Domain 2: Deviations from Intended Interventions	Domain 3: Missing Outcome Data	Domain 4: Measurement of Outcome	Domain 5: Selection of Reported Result	Overall Risk of Bias
Boyle et al. [29]						
Dotterud et al. [30]						
Huurre et al. [31]						
Rautava et al. [32]						
Simpson et al. [33]						
Wickens et al. [34]						

**Table 4:** Quality assessment of included studies based on the Jadad quality assessment.

Reference	Was the Study Described as Randomised?	Was the Method Used to Generate the Sequence of Randomisation Described and Appropriate?	Was the Study Described as Double-Blind?	Was There a Description of the Withdrawals and Drop-Outs?	Deduct one Point If the Method Used to Generate the Sequence of Randomisation Was Described and It Was Inappropriate	Deduct One Point If the Study Was Described as Double-Blind but the Method Blinding Was Inappropriate	Jadad Score (1 to 5)
Boyle et al. [29]	1	1	1	1	0	0	5
Dotterud et al. [30]	1	1	1	1	0	0	5
Huurre et al. [31]	1	0	1	1	0	0	3
Rautava et al. [32]	1	1	1	1	0	0	5
Simpson et al. [33]	1	1	1	1	0	0	5
Wickens et al. [34]	1	1	1	1	0	0	5

## Data Availability

Not applicable.
